# Automated item‐level measures of verbal fluency in semantic and logopenic primary progressive aphasia

**DOI:** 10.1002/alz.71124

**Published:** 2026-01-19

**Authors:** Jet M. J. Vonk, Franco J. Ferrante, Brittany T. Morin, Diana Alejandra Rodriguez, Mia Lin, Rian Bogley, Jessica de Leon, Boon Lead Tee, Miguel Ángel Santos‐Santos, Zachary A. Miller, Maria Luisa Mandelli, Maria Luisa Gorno‐Tempini, Adolfo M. García

**Affiliations:** ^1^ Department of Neurology Edward and Pearl Fein Memory and Aging Center University of California San Francisco San Francisco California USA; ^2^ Centro de Neurociencias Cognitivas Universidad de San Andrés Victoria Buenos Aires Argentina; ^3^ Department of Neurology Sant Pau Memory Unit Hospital de la Santa Creu I Sant Pau ‐ Sant Pau Biomedical Research Institute Barcelona Spain; ^4^ Network Center for Biomedical Research in Neurodegenerative Diseases (CIBERNED) National Institute of Health Carlos III Madrid Spain; ^5^ Global Brain Health Institute University of California San Francisco San Francisco California USA; ^6^ Departamento de Lingüística y Literatura Facultad de Humanidades Universidad de Santiago de Chile Santiago Chile

**Keywords:** category fluency, digital biomarkers, frontotemporal dementia, language assessment, lexical access, psycholinguistic, semantic memory, word properties

## Abstract

**INTRODUCTION:**

Verbal fluency tasks are widely used in primary progressive aphasia (PPA), but most studies rely only on total correct responses, overlooking qualitative features of the words produced. We applied a scalable computational framework to extract item‐level features from fluency responses in semantic variant (svPPA) and logopenic variant PPA (lvPPA) to test their value for differential diagnosis.

**METHODS:**

We analyzed animal fluency responses from 113 participants (40 svPPA, 40 lvPPA, 33 controls) using an automated pipeline extracting nine psycholinguistic features. Group differences were examined with (co)variance models, classification with logistic regression, and brain–behavior associations via structural magnetic resonance imaging.

**RESULTS:**

All features except semantic variability distinguished svPPA from lvPPA. Models including features outperformed (area under the curve [AUC] = 0.86) those using only total correct or clinical variables (AUC = 0.60–0.68). Features related mainly to temporal lobe atrophy, whereas total correct also related to the angular gyrus.

**DISCUSSION:**

Automated item‐level analysis offers a sensitive, scalable method for supporting PPA diagnosis and monitoring.

**Highlights:**

- Automated item‐level features from verbal fluency aid semantic variant primary progressive aphasia (PPA) versus logopenic variant PPA diagnosis.
- Item‐level fluency features outperform total correct responses for classification.
- Item‐level fluency features map onto syndrome‐relevant temporal lobe atrophy.
- A scalable, fully automated pipeline enables integration into clinical practice.
- There is potential to support low‐burden, objective monitoring of disease progression in PPA.

## 1 BACKGROUND

Primary progressive aphasia (PPA) encompasses a group of neurodegenerative disorders characterized by progressive speech and language impairment.^1^, ^2^ Among its variants, semantic variant PPA (svPPA) is defined by a breakdown in conceptual knowledge and word comprehension, typically linked to anterior temporal lobe atrophy, particularly in the left hemisphere.^3^, ^4^ In contrast, logopenic variant PPA (lvPPA) is primarily characterized by impairments in phonological short‐term memory and sentence repetition, with atrophy observed in the posterior temporal and inferior parietal regions.^5^ Despite these distinct profiles, both syndromes affect word retrieval and speech output^6^, ^7^ and can present with overlapping or evolving features.^8^, ^9^, ^10^ While experienced clinicians using comprehensive batteries can reliably distinguish these syndromes (at least, in English speakers^11^, ^12^, ^13^, ^14^), less experienced clinicians may face challenges differentiating variants, especially when time constraints limit comprehensive testing. Beyond these practical considerations, emerging computational methods offer powerful ways to enhance existing assessments by extracting meaningful linguistic markers that reflect the known semantic and phonological distinctions between svPPA and lvPPA. Automated analysis can uncover these fine‐grained language patterns from tasks already embedded in standard neuropsychological practice, increasing their diagnostic and theoretical value without adding clinical burden. Such computational refinements increase the informational yield of standard assessments and lay the groundwork for scalable, reproducible tools that link linguistic performance to disease‐specific processes.

Recent advances in computational linguistics and artificial intelligence have enabled automated extraction of word properties from verbal fluency tasks,^15^, ^16^ offering a more detailed, objective, and scalable alternative to manual scoring methods. These methods allow systematic analysis of item‐level linguistic features, providing insights into the neural mechanisms underlying speech and language, as well as cognitive deficits in PPA.^17^, ^18^, ^19^, ^20^ The animal (category) verbal fluency task, wherein individuals generate as many animal names as possible within 1 minute, is widely used in neuropsychological assessments.^21^ Traditionally, performance is quantified by total correct responses, which provides a general measure of semantic memory and executive function. However, this approach may obscure important qualitative differences in the words generated, overlooking potentially useful information for diagnosis and profiling.^17^, ^22^

Item‐level analysis of verbal fluency responses—examining word properties such as lexical frequency and age of acquisition—can offer deeper insights into the mechanisms underlying performance.^23^ Studies suggest that item‐level metrics may better capture the multidimensional nature of verbal fluency deficits.^24^ To date, most item‐level analyses have been limited by the labor‐intensive nature of manual coding, reducing scalability in clinical practice.^16^ Automated approaches now allow rapid and objective extraction of these features, making item‐level analysis feasible for large‐scale applications.^25^ While studies in other neurodegenerative disorders—including those with semantic memory deficits—have demonstrated the utility of word property‐based approaches in detecting early cognitive changes,^15^, ^26^, ^27^, ^28^, ^29^ this methodology is less widely applied to PPA,^17^ and rarely with automation. For example, Alzheimer's disease, which affects semantic memory, shows alterations in lexical frequency, semantic granularity, and phonological neighborhood density, and can be more accurately distinguished from behavioral variant frontotemporal dementia using word properties than total correct responses.^15^ The question remains whether a multivariate analysis of item‐level verbal fluency characteristics can offer diagnostic insight beyond total correct responses in distinguishing svPPA and lvPPA; a detailed examination of these linguistic features may help address the diagnostic challenge posed by overlapping symptoms.

This study investigated whether item‐level features of verbal fluency differ among svPPA, lvPPA, and healthy controls, and whether these differences can improve PPA syndrome identification. Given the profound semantic impairment in svPPA,^3^ we hypothesized that these individuals produce words with lower semantic granularity, lower age of acquisition, and other semantic features, while those with lvPPA—given the phonological and lexical nature of their impairment^6^—show impairments in lexical features such as phonological neighborhood density and syllable/phoneme length. Additionally, we expected these linguistic features to correlate with distinct neuroanatomical atrophy patterns. As the angular and supramarginal gyri—commonly atrophied in lvPPA—are linked to phonological processing and lexical access, we hypothesized that lexical features should associate with posterior temporal and inferior parietal regions. In contrast, anterior temporal structures—particularly the left temporal pole—play a central role in semantic memory and conceptual knowledge; thus, we hypothesized semantic features to associate with anterior temporal regions.

## 2 METHODS

### 2.1 Participants

This study included 113 participants from the University of California, San Francisco (UCSF) Edward and Pearl Fein Memory and Aging Center (MAC): 40 with svPPA, 40 with lvPPA, and 33 cognitively healthy controls (Table 1). The UCSF Fein MAC enrolls individuals for research referred for evaluation due to cognitive, behavioral, or language concerns, undergoing comprehensive clinical, neuropsychological, and imaging assessments. Healthy control participants were primarily recruited through the Brain Aging Network for Cognitive Health (BrANCH) and were screened for neurological and psychiatric conditions to confirm their cognitive health.

**TABLE 1 alz71124-tbl-0001:** Participant characteristics (m [SD] for continuous variables and *n* [%] for categorial variables); *p* value across all groups.

	Healthy (n = 33)	svPPA (n = 40)	lvPPA (n = 40)	*p* value
Age	68.85 (5.7, 49–75)	65.75 (7, 50–83)	65.83 (8.7, 51–82)	0.073
Sex/gender (women)	21 (63.6%)	16 (40%)	21 (52.5%)	0.130
Education (years)	17.42 (2.4, 12–20)	17.12 (2.4, 12–22)	17.2 (3.1, 12–23)	0.886
Race/ethnicity				
White	33 (100%)	37 (92.5%)	38 (95%)	0.465
Chinese	0 (0%)	1 (2.5%)	0 (0%)	
Unknown/refused to state	0 (0%)	2 (5%)	2 (5%)	
Handedness				
Right	30 (90.9%)	34 (85%)	34 (85%)	0.630
Left	3 (9.1%)	3 (7.5%)	4 (10%)	
Ambidextrous	0 (0%)	3 (7.5%)	2 (5%)	
MMSE	29.48 (0.7, 28–30)	25.55 (3.5, 16–30)	24.15 (3.3, 17–30)	<0.001
CDR Sum of Boxes	0 (0, 0–0)	2.27 (2.2, 0–9.5)	1.74 (1.4, 0–4.5)	<0.001

Abbreviations: CDR, Clinical Dementia Rating; lvPPA, logopenic variant primary progressive aphasia; MMSE, Mini‐Mental State Examination; SD, standard deviation; svPPA, semantic variant primary progressive aphasia

For sample selection, we identified all individuals with a clinical diagnosis of svPPA or lvPPA based on established diagnostic criteria,^3^ who had research visits in the UCSF Fein MAC database. For participants with Neary‐Semantic and svPPA, diagnostic charts confirmed predominant left‐lateralized atrophy. Participants were excluded if they did not complete the verbal fluency task, were not native English speakers, had predominantly right‐lateralized atrophy as described in their chart, did not have a Mini‐Mental State Examination (MMSE)^30^ score, had an MMSE score < 15 for the PPA groups or < 30 for the healthy control group, or had a Clinical Dementia Rating (CDR)^31^ score > 0 in the control group. To minimize confounding factors and account for differences in subgroup sizes, we performed frequency matching to match groups on age, sex/gender, and education, while ensuring the svPPA and lvPPA groups were also matched on MMSE scores (*t*[78] = 1.84, *p* = 0.070) and CDR Sum of Boxes scores (*t*[74] = 1.27, *p* = 0.207). All participants or their caregivers provided informed consent in accordance with the Declaration of Helsinki. The study was approved by the UCSF Institutional Review Board (IRB #10‐03946) in accordance with the ethical standards as laid down in the 1964 Declaration of Helsinki and its later amendments.

**RESEARCH IN CONTEXT**

1. **Systematic review**: We searched PubMed and Google Scholar for studies published up to March 2025 using the terms “primary progressive aphasia” (PPA), “semantic variant,” “logopenic variant,” “verbal/animal/category fluency,” “word properties,” and “item‐level analysis.” Prior research has shown that verbal fluency performance is typically scored by total correct responses, with limited use of item‐level linguistic features due to the labor‐intensive nature of manual coding. Although studies in Alzheimer's disease and other dementias have suggested diagnostic value of word property analyses, evidence in PPA remains scarce.
2. **Interpretation**: Our findings show that automated extraction of word properties from a standard fluency task distinguishes semantic from logopenic variant PPA more accurately than total correct scores. Word properties were linked to left temporal regions, but not to parietal regions.
3. **Future directions**: Future studies should validate these findings longitudinally, in larger, more diverse samples, and assess their utility for disease monitoring and early detection.

### 2.2 Verbal fluency task and extraction of word properties

Participants completed a verbal fluency task in which they were instructed to generate as many animals as possible within 60 seconds. The examiner recorded responses on paper in real time, and responses were later digitized and checked for accuracy. Participants were instructed to avoid repetitions, proper names, and morphological variants of the same word. In addition to the standard fluency metric (total number of correct responses), we analyzed word properties using an automated extraction approach via the Toolkit to Examine Lifelike Language (TELL).^15^, ^32^, ^33^ Each word was characterized along nine linguistic dimensions: number of phonemes, number of syllables, phonological neighborhood size, lexical frequency, age of acquisition, semantic neighborhood density, word concreteness, semantic variability, and semantic granularity.[Table alz71124-tbl-0001]

Lexical frequency data were sourced from SUBTLEXus, which comprises 51 million words from American English film subtitles (spanning 1900–2007) and television subtitles (with an unspecified timeframe).^34^ Age of acquisition metrics were obtained from norms encompassing > 30,000 English words, based on subjective assessments in which participants indicated the age at which they believed they learned each word.^35^ This dataset also includes information on the number of phonemes and syllables for each word. Semantic neighborhood density was derived from the co‐occurrence matrix of 287,000 word pairs,^36^ accessible via the English Lexicon Project.^37^ Sequential metrics of clusters and switches were calculated using the Semantic Network and Fluency Utility (SNAFU), an automated method for identifying clusters and switches in animal verbal fluency.^38^ Word concreteness ratings were sourced from a norming study involving > 4000 participants, who provided subjective ratings on a scale from 1 to 5 for 37,058 English words.^39^ Data collection was conducted through internet crowdsourcing. Phonological neighborhood information was obtained from Clearpond, calculated as the number of words resulting from substituting, adding, or omitting a phoneme in a set of 27,751 English words.^40^ We assessed each word's semantic granularity using Python's Natural Language Toolkit (NLTK)^41^ to interface with WordNet, a hierarchical lexical database that organizes words from general categories to specific concepts. In this structure, “entity” is the most general category, with subsequent nodes becoming increasingly specific (e.g., “animal” to “dog” to “bulldog”). We defined semantic granularity by counting the nodes between a word and the “entity” root; thus, words in bin‐3 are closer to “entity” than those in bin‐10, indicating that the former are more general concepts.^15^, ^42^

Finally, we assessed semantic variability by using the English FastText model (cc.en.300.bin) to generate vector representations for each word. This 300‐dimensional FastText model is pre‐trained on a mixture of May 2017 Common Crawl and September 11, 2017 Wikipedia data.^43^ FastText represents each word as a combination of vectors for its smaller character fragments (e.g., prefixes and suffixes), producing morphology‐aware embeddings that remain robust even for rare or misspelled words. In this vector space, words that appear in similar contexts are positioned close together, allowing cosine distance between vectors to serve as a reliable measure of semantic similarity.^44^ We then calculated semantic variability as the variance in semantic distance between consecutive words; a higher variance suggests less consistent semantic relationships across the word sequence.^42^, ^45^ The psycholinguistic metrics of lexical frequency, age of acquisition, number of phonemes, number of syllables, concreteness, phonological neighborhood density, and semantic neighborhood density are available from existing and freely accessible linguistic corpora; spreadsheets with those metrics used in this study are also available for download at https://github.com/jmjvonk.^28^

To fully capture the distributional properties of each feature, we extracted seven statistical descriptors for each metric—mean, median, standard deviation, minimum, maximum, skewness, and kurtosis—except for semantic variability, which was calculated as a single value based on the entire set of words rather than individual words. To ensure consistency across participants, all words were processed using an automated pipeline, and missing values were handled according to standardized imputation procedures when necessary.^45^, ^46^

### 2.3 Statistical analysis

We conducted descriptive statistical analyses to examine sample characteristics and feature distributions, using general linear models, chi‐squared tests, and Pearson correlation coefficients. Correlations among word properties were assessed using a Pearson correlation matrix in the cognitively healthy control group.

To evaluate group differences in verbal fluency metrics (i.e., word properties and total correct), we performed analysis of (co)variance models, both unadjusted (ANOVA) and adjusted for covariates (ANCOVA). We standardized verbal fluency metrics across the whole sample (svPPA, lvPPA, controls) to ease comparison of estimates across the different metrics. Post hoc pairwise comparisons were conducted for significant effects, with multiple comparisons controlled using the Benjamini–Hochberg procedure's false discovery rate (FDR) correction. Covariates included age, education, sex/gender, and disease severity. In additional models, we also adjusted for total correct score in the models for word properties. Disease severity was calculated as a composite score of the CDR Sum of Boxes and MMSE, following previous work.^20^ Both scores were scaled from 0 to 1 based on the sample's minimum and maximum values, with MMSE reversed; the scaled scores were averaged, yielding a 0 to 1 disease severity index in which higher values indicate greater disease severity. One participant with lvPPA and three participants with svPPA were missing CDR Sum of Boxes scores; in those cases, the scaled score on the MMSE represented disease severity.

For classification, we implemented binary logistic regression models to distinguish svPPA from lvPPA. We examined five models, including (1) only word properties and total correct; (2) word properties, total correct, and covariates; (3) only covariates; (4) only total correct; and (5) total correct and covariates. Each model's classification performance was evaluated using the area under the receiver operating characteristic curve (AUC) with 95% confidence intervals and confusion matrix (sensitivity, specificity). Pairwise comparisons of AUC values were conducted using the DeLong test to assess differences between models.

All statistical analyses were conducted in R (version 4.3.0). Analyses, figures, and tables were generated using R packages including caret, MASS, pROC, dplyr, ggplot2, GGally, stringr, foreign, RColorBrewer, car, multcomp, emmeans, tidyr, gt, furniture, epitools, and reshape2. All analysis code is available at https://github.com/jmjvonk. The conditions of our ethics approval do not allow public archiving of anonymized study data. Data generated by the UCSF Fein MAC are available upon request and subject to approval. Requests should be submitted through the UCSF Fein MAC Resource Request form: http://memory.ucsf.edu/resources/data. Access is granted to named researchers following UCSF's ethical procedures for data sharing, which require submission of a material transfer agreement (MTA) available at https://icd.ucsf.edu/material‐transfer‐and‐data‐agreements. Data are not available for commercial use.

### 2.4 Neuroimaging analysis

Within our sample, 62 participants had a brain magnetic resonance imaging (MRI) scan within 1 year of their verbal fluency task, including 18 lvPPA, 23 svPPA, and 21 healthy controls. We conducted analyses using the Computational Anatomy Toolbox 12 (dbm.neuro.uni‐jena.de/cat) within the Statistical Parametric Mapping 12 (SPM12) software (fil.ion.ucl.ac.uk/spm/software/spm12), running in MATLAB R2024b. High‐resolution T1‐weighted MRI scans were acquired for all participants using previously described sequences on either 1.5T (*n* = 19),^2^ 3T (*n* = 40),^47^ or 4T (*n* = 3)^48^ scanners. After a visual quality check, structural images were preprocessed with a spatially adaptive non‐local means denoising algorithm and bias field correction to enhance image quality. The images were then aligned via affine transformation and processed using SPM12's “unified segmentation” approach. Next, they were (1) segmented into gray matter, white matter, and cerebrospinal fluid; (2) spatially normalized to the Montreal Neurological Institute (MNI) space using an advanced geodesic shooting technique; (3) modulated by Jacobian determinants to preserve tissue volume; (4) resampled to an isotropic voxel size of 1.5 × 1.5 × 1.5 mm^3^; and (5) smoothed with an 8 mm full‐width at half‐maximum (FWHM) Gaussian kernel to account for residual anatomical variability.

We estimated gray matter volume to assess atrophy patterns in each patient group compared to healthy controls using voxel‐based morphometry (VBM) whole‐brain analyses with ANOVAs, controlling for total intracranial volume (TIV), age, sex/gender, and scanner type as nuisance variables. Alpha levels after family‐wise error (FWE) corrections were set at *p* < 0.05 with a minimum cluster size of 100 voxels. Visualization was performed using xjView toolbox (https://www.alivelearn.net/xjview). To examine brain–behavior relationships, we tested associations between values of the linguistic features and gray matter volume loss within regions of interest (ROIs),^49^ using the Neuromorphometrics atlas (http://Neuromorphometrics.com/). We conducted linear regressions with TIV, age, sex/gender, and scanner type as covariates across the entire sample (i.e., control, svPPA and lvPPA together) with brain MRI (*n* = 62). We report original *p* values as well as *p* values adjusted for multiple comparisons using the Benjamini–Hochberg procedure's FDR. Targeted ROIs were selected based on established atrophy patterns in lvPPA and svPPA, focusing on key left hemisphere regions: the temporal pole (specific to svPPA), angular gyrus (specific to lvPPA), supramarginal gyrus (specific to lvPPA), middle temporal gyrus (atrophied in both groups), and superior temporal gyrus (atrophied in both groups). ANCOVA model results (adjusted for age, sex/gender, TIV, and scanner type) confirmed no significant differences in mean atrophy between svPPA and lvPPA groups in the middle temporal gyrus (*F*[1,35] = 1.85, *p* = 0.182) and superior temporal gyrus (*F*[1,35] = .50, *p* = 0.484), whereas the syndrome‐specific ROIs showed greater atrophy in the corresponding patient group—temporal pole in svPPA and angular/supramarginal gyrus in lvPPA (all *p* < 0.005).

## 3 RESULTS

### 3.1 Behavioral

Sample characteristics for demographic and cognitive variables across groups are summarized in Table 1. As groups were matched on age, sex/gender, and education, there were no significant differences among groups in these variables. The cognitively healthy control group had higher MMSE and lower CDR Sum of Boxes scores than both PPA groups, while the svPPA and lvPPA groups did not significantly differ from each other in MMSE (*t*[78] = 1.840, *p *= 0.070) or CDR Sum of Boxes (*t*[74] = 1.272, *p* = 0.207).

**FIGURE 1:**
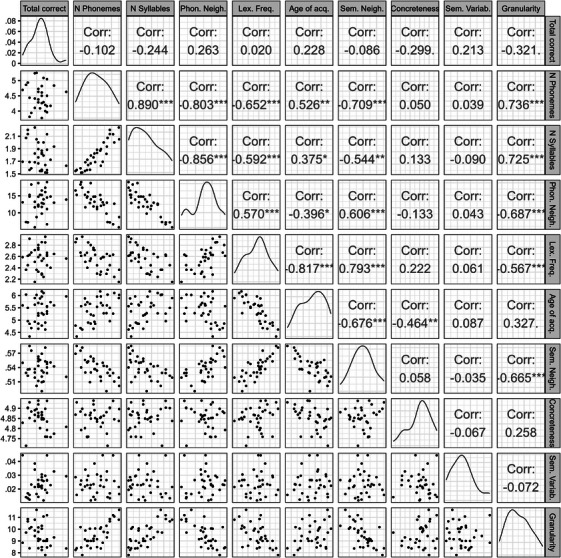
Correlation matrix of verbal fluency metrics including scatterplots and distributions. * *p* < 0.05, ** *p* < 0.01, *** *p* < 0.001. Age of acq., age of acquisition; Corr., correlation; Lex. frequency, lexical frequency; *N*, number; Phon. Neigh, phonological neighborhood density; Sem. Neigh., semantic neighborhood density; Sem. Variab., semantic variability.

In the cognitively healthy control group, correlations among word properties and total number of correct responses were examined using a Pearson correlation matrix (Figure 1). Total number of correct responses was weakly correlated with word properties (*r* = 0.02–0.03). Semantic variability and concreteness were not strongly associated with other word properties (except for concreteness with age of acquisition). Several word property metrics showed moderate to strong correlations, which were particularly strong (*r* > 0.80) among the group of phonological/lexical properties (number of phonemes, number of syllables, phonological neighborhood density) and moderately strong (*r* > 0.65) among properties considered to be related to both lexical and semantic aspects of words^28^ (lexical frequency, age of acquisition, semantic neighborhood density). The semantic property of semantic granularity was moderately to strongly correlated with both lexical and lexical/semantic properties.

**FIGURE 2:**
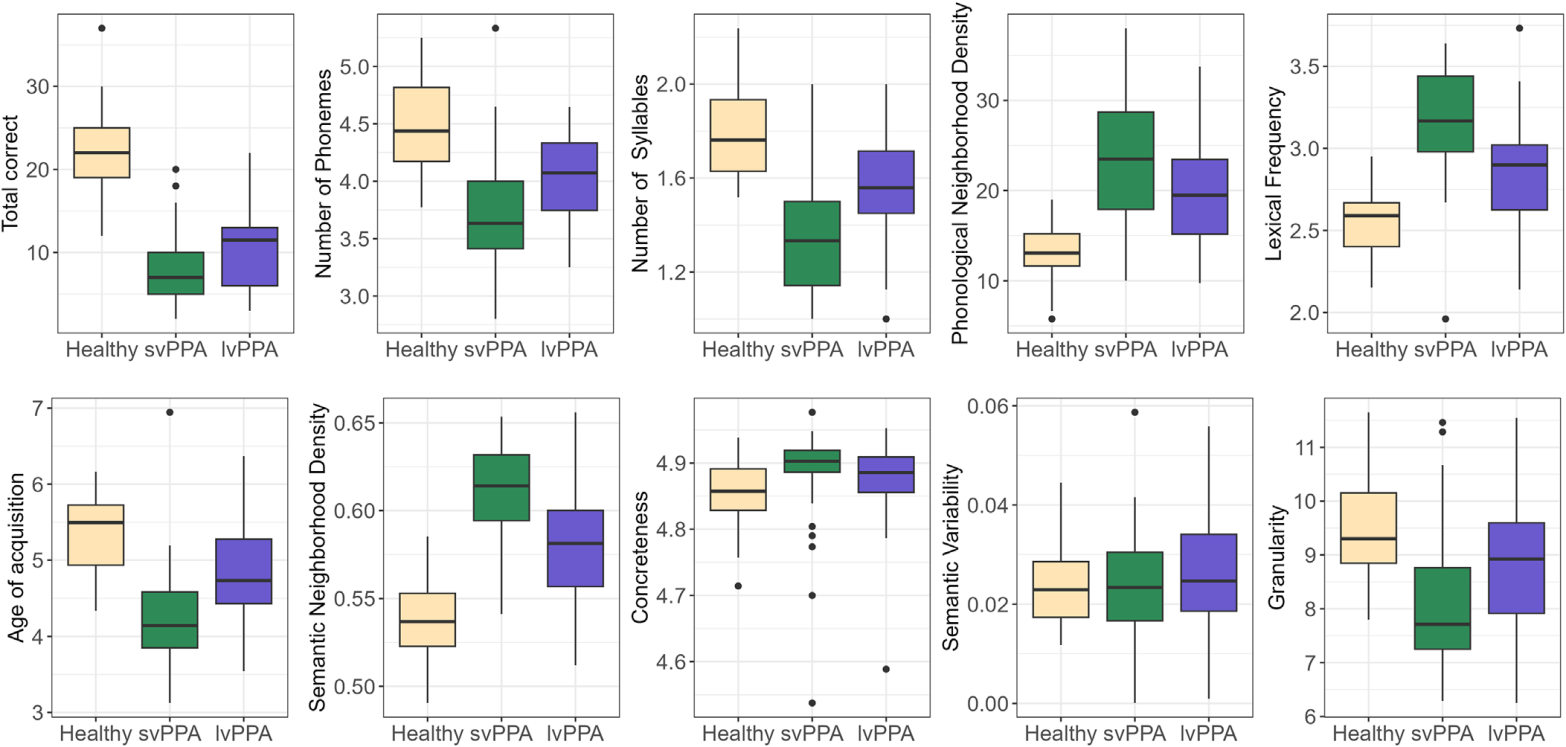
Performance on fluency metrics across diagnostic groups (healthy *n* = 33; svPPA *n* = 40; lvPPA *n* = 40). lvPPA, logopenic variant primary progressive aphasia; svPPA, semantic variant primary progressive aphasia.

AN(C)OVA models to assess group differences in verbal fluency metrics showed similar results unadjusted or adjusted for covariates (age, sex/gender, education, disease severity), except for concreteness, which became significant across groups in the adjusted models; this report focuses on the adjusted models. All word properties and total correct differed across diagnostic groups, except for semantic variability. Pairwise comparisons (FDR corrected) showed differences between the lvPPA and svPPA groups on every metric except concreteness and semantic variability (Table 2). The svPPA group differed from the control group on each metric except semantic variability (9 out of 10). Meanwhile, the lvPPA group differed from the control group on fewer metrics (6 out of 10), yet with differences in number of phonemes and syllables, phonological neighborhood density, semantic neighborhood density, concreteness, and total correct. The direction of differences between groups is visualized in the boxplots in Figure 2. In models of word properties that additionally adjusted for total correct score, the overall pattern of results remained consistent with the models unadjusted for total score, particularly for comparisons between the svPPA and lvPPA groups (Table 2).

**TABLE 2:** Pairwise analysis of covariance comparisons of verbal fluency metrics across diagnostic groups adjusted for age, sex/gender, education, and disease severity, as well as with and without adjustment for total correct.

		Without adjustment for total correct score						With adjustment for total correct score					
Dependent variable	Contrast	Estimate	SE	df	*t* value	*p* value	*p* value (FDR)	Estimate	SE	df	*t* value	*p* value	*p* value (FDR)
Total correct	Healthy—lvPPA	1.192	0.17	106	6.97	<0.001	<.001	–	–	–	–	–	–
N Phonemes		0.975	0.25	106	3.90	<0.001	<0.001	0.638	0.30	105	2.14	0.034	0.061
N Syllables		0.671	0.24	106	2.75	0.007	0.010	0.375	.29	105	1.29	0.202	0.275
Phon. Neigh.		−0.799	0.25	106	−3.22	0.002	0.003	−0.366	0.29	105	−1.26	0.212	0.277
Lex. Freq.		−0.423	0.23	106	−1.86	0.066	0.083	0.069	0.26	105	0.26	0.792	0.820
Age of Acq.		0.360	0.23	106	1.57	0.118	0.142	−0.221	0.26	105	−0.86	0.394	0.461
Sem. Neigh.		−0.778	0.21	106	−3.77	<0.001	0.001	−0.339	0.24	105	−1.42	0.159	0.227
Concreteness		−0.799	0.30	106	−2.63	0.010	0.013	−0.426	0.36	105	−1.17	0.243	0.304
Sem. Variab.		−0.345	0.30	106	−1.14	0.257	0.297	−0.639	0.36	105	−1.76	0.082	0.137
Semantic granularity		0.102	0.28	106	0.37	0.713	0.737	0.238	0.34	105	0.71	0.478	0.532
Total correct	Healthy ‐ svPPA	1.624	0.17	106	9.70	<0.001	<0.001	–	–	–	–	–	–
N Phonemes		1.587	0.24	106	6.48	<0.001	<.001	1.127	0.33	105	3.40	0.001	0.003
N Syllables		1.427	0.24	106	5.97	<0.001	<0.001	1.024	0.33	105	3.15	0.002	0.006
Phon. Neigh.		−1.420	0.24	106	−5.84	<0.001	<0.001	−0.831	0.33	105	−2.55	0.012	0.028
Lex. Freq.		−1.341	0.22	106	−6.02	<0.001	<0.001	−0.671	0.29	105	−2.29	0.024	0.045
Age of Acq.		1.244	0.22	106	5.55	<0.001	<0.001	0.451	0.29	105	1.57	.120	0.190
Sem. Neigh.		−1.640	0.20	106	−8.11	<0.001	<.001	−1.042	0.27	105	−3.92	<0.001	0.001
Concreteness		−0.850	0.30	106	−2.86	0.005	0.008	−0.342	0.40	105	−0.85	0.399	0.461
Sem. Variab.		−0.199	0.30	106	−0.67	0.503	0.550	−0.600	0.41	105	−1.48	0.142	0.213
Semantic granularity		0.731	0.27	106	2.70	0.008	0.011	0.917	0.37	105	2.46	0.016	0.031
Total correct	lvPPA ‐ svPPA	0.432	0.13	106	3.45	0.001	0.002	–	–	–	–	–	–
N Phonemes		0.611	0.18	106	3.34	0.001	0.002	0.489	0.19	105	2.57	0.012	0.028
N Syllables		0.756	0.18	106	4.22	<0.001	<0.001	0.649	0.19	105	3.47	0.001	0.003
Phon. Neigh.		−0.621	0.18	106	−3.41	0.001	0.002	−0.464	0.19	105	−2.48	0.015	0.031
Lex. Freq.		−0.919	0.17	106	−5.50	<0.001	<0.001	−0.740	0.17	105	−4.40	<0.001	<0.001
Age of Acq.		0.884	0.17	106	5.26	<0.001	<0.001	0.673	0.17	105	4.06	<0.001	0.001
Sem. Neigh.		−0.863	0.15	106	−5.70	<0.001	<0.001	−0.703	0.15	105	−4.60	<0.001	<0.001
Concreteness		−0.051	0.22	106	−0.23	0.818	0.818	0.084	0.23	105	0.36	0.718	0.769
Sem. Variab.		0.146	0.22	106	0.66	0.514	0.550	0.039	0.23	105	0.17	0.868	0.868
Semantic granularity		0.629	0.20	106	3.10	0.002	0.004	0.679	0.21	105	3.16	0.002	0.006

*Note*. Verbal fluency metrics are standardized on the mean and standard deviation of the whole sample.

Abbreviations: Age of acq., age of acquisition; FDR, false discovery rate; Lex. frequency, lexical frequency; lvPPA, logopenic variant primary progressive aphasia; N, number; Phon. Neigh., phonological neighborhood density; SE, standard error; Sem. Neigh., semantic neighborhood density; Sem. Variab., semantic variability; svPPA, semantic variant primary progressive aphasia.

**FIGURE 3:**
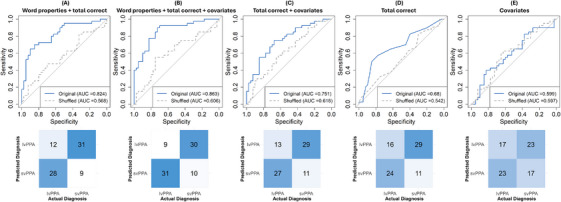
Receiver operating characteristic curves and confusion matrices for diagnostic classification of lvPPA and svPPA to assess model performance and validation through diagnostic label shuffling. AUC, area under the receiver operating characteristic curve; lvPPA, logopenic variant primary progressive aphasia; svPPA, semantic variant primary progressive aphasia.

Logistic regression models to classify svPPA versus lvPPA showed that models with word properties outperform their counterparts without these variables, as confirmed by statistical comparisons of AUCs between models with and without these features. A model with word properties and total correct achieved an AUC of .83 (95% confidence interval [CI]: 0.73, 0.92). Adding covariates (i.e., age, sex/gender, education, disease severity) to this model of word properties and total correct slightly improved performance (not significantly; *z* = −1.16, *p* = 0.245), yielding an AUC of .86 (95% CI: 0.78, 0.94). A model using only total correct with an AUC of .68 (95% CI: .56, .80) was less effective than word properties + total correct in classification (*z* = 2.82, *p* = 0.005); adding covariates to this model of total correct slightly improved performance with an AUC of 0.75 (95% CI: 0.64, 0.86). A model including only covariates had the lowest performance with an AUC of .60 (95% CI: 0.47, 0.73), underperforming compared to the models of covariates with total correct (*z* = −2.16, *p* = 0.031) and covariates with total correct and word properties (*z* = 2.64, *p* = 0.008). Validation through diagnostic label shuffling resulted in near‐chance classification, supporting the robustness of these features in distinguishing between svPPA and lvPPA rather than reflecting random variation in the sample. Figure 3 presents a visualization of receiver operating characteristic = curves and confusion matrices.

### 3.2 Neuroimaging

**FIGURE 4:**
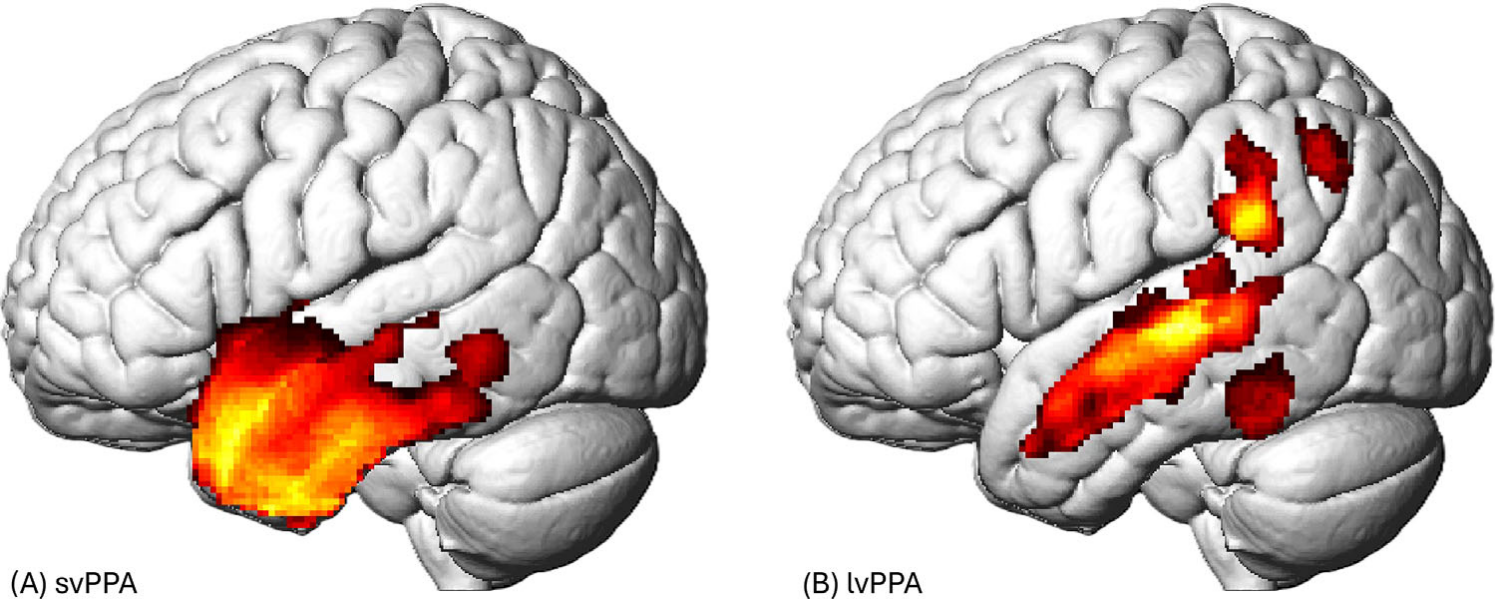
Structural atrophy patterns in (A) semantic variant primary progressive aphasia (svPPA) and (B) logopenic variant primary progressive aphasia (lvPPA).

Participants in the svPPA group exhibited predominantly left‐sided gray matter atrophy in the left temporal pole; left inferior, middle, and superior temporal gyri; left fusiform gyrus; and left insula, as well as some atrophy in the right temporal pole (*p* < 0.05 FWE corrected; Figure 4A). The lvPPA group showed left‐lateralized gray matter atrophy in the angular gyrus, supramarginal gyrus, and left inferior, middle, and superior temporal gyri (*p* < 0.05 FWE corrected; Figure 4B).

Brain–behavior analyses revealed several associations between values on linguistic features and gray matter volume across ROIs; brain–behavior results are fully detailed in Table 3. Overall, lower semantic granularity, fewer number of phonemes and syllables, higher phonological and semantic neighborhood density, higher lexical frequency, and earlier age of acquisition were associated with lower volume in the left temporal pole, left middle temporal gyrus, and left superior temporal gyrus (all FDR corrected *p* < 0.05, except phonological neighborhood density with the left superior temporal gyrus at *p* = 0.073 FDR corrected). Fewer number of total correct responses was associated with less volume in the left angular gyrus, left temporal pole, left middle temporal gyrus, and left superior temporal gyrus (all FDR corrected *p* < 0.05). No other associations were observed between fluency metrics and the left angular or left supramarginal gyri. Concreteness and semantic variability were not associated with any of the five ROIs.

**TABLE 3 alz71124-tbl-0003:** Brain‐behavior correlations between selected regions of interest and verbal fluency metrics.

Brain region of interest	Verbal fluency metric	Estimate	Standard error	*t* value	*p* value	*p* value (FDR)
Left temporal pole	Semantic granularity	0.0773	0.014	5.63	<0.001	<0.001
	Total correct	0.0822	0.012	6.88	<0.001	<0.001
	Number of phonemes	0.0798	0.014	5.75	<0.001	<0.001
	Number of syllables	0.0820	0.014	5.86	<0.001	<0.001
	Phonological neighborhood density	−0.0689	0.015	−4.75	<0.001	<0.001
	Lexical frequency	−0.0877	0.012	−7.06	<0.001	<0.001
	Age of acquisition	0.0859	0.012	7.23	<0.001	<0.001
	Semantic neighborhood density	−0.0816	0.012	−6.86	<0.001	<0.001
	Concreteness	−0.0121	0.014	−0.85	0.398	0.530
	Semantic variability	0.0190	0.018	1.08	0.286	0.445
Left middle temporal gyrus	Semantic granularity	0.0495	0.014	3.57	0.001	0.002
	Total correct	0.0592	0.012	4.87	<0.001	<0.001
	Number of phonemes	0.0533	0.014	3.82	<0.001	0.001
	Number of syllables	0.0539	0.014	3.81	<0.001	0.001
	Phonological neighborhood density	−0.0399	0.014	−2.77	0.008	0.018
	Lexical frequency	−0.0577	0.013	−4.37	<0.001	<0.001
	Age of acquisition	0.0594	0.012	4.78	<0.001	<0.001
	Semantic neighborhood density	−0.0536	0.013	−4.29	<0.001	<0.001
	Concreteness	−0.0153	0.013	−1.21	0.231	0.440
	Semantic variability	0.0079	0.016	0.50	0.619	0.774
Left supramarginal gyrus	Semantic granularity	0.0012	0.013	0.09	0.927	0.951
	Total correct	0.0128	0.012	1.08	0.285	0.445
	Number of phonemes	−0.0049	0.013	−0.38	0.704	0.786
	Number of syllables	−0.0050	0.013	−0.38	0.704	0.786
	Phonological neighborhood density	0.0048	0.013	0.38	0.708	0.786
	Lexical frequency	0.0000	0.013	0.00	0.997	0.997
	Age of acquisition	0.0051	0.012	0.42	0.680	0.786
	Semantic neighborhood density	−0.0033	0.012	−0.27	0.785	0.826
	Concreteness	−0.0102	0.010	−0.98	0.333	0.475
	Semantic variability	−0.0152	0.013	−1.17	0.246	0.445
Left angular gyrus	Semantic granularity	0.0067	0.010	0.70	0.486	0.627
	Total correct	0.0248	0.008	2.93	0.005	0.012
	Number of phonemes	0.0099	0.010	1.02	0.313	0.464
	Number of syllables	0.0105	0.010	1.07	0.289	0.445
	Phonological neighborhood density	−0.0084	0.010	−0.88	0.382	0.526
	Lexical frequency	−0.0104	0.009	−1.10	0.275	0.445
	Age of acquisition	0.0151	0.009	1.67	0.101	0.224
	Semantic neighborhood density	−0.0109	0.009	−1.22	0.228	0.440
	Concreteness	−0.0109	0.008	−1.38	0.172	0.362
	Semantic variability	0.0028	0.010	0.280	0.780	0.826
Left superior temporal gyrus	Semantic granularity	0.0331	0.010	2.66	0.010	0.021
	Total correct	0.0471	0.010	4.38	<0.001	<0.001
	Number of phonemes	0.0368	0.010	2.94	0.005	0.011
	Number of syllables	0.0376	0.010	2.96	0.004	0.011
	Phonological neighborhood density	−0.0272	0.010	−2.14	0.037	0.073
	Lexical frequency	−0.0361	0.010	−2.96	0.005	0.011
	Age of acquisition	0.0400	0.010	3.48	0.001	0.003
	Semantic neighborhood density	−0.0394	0.010	−3.50	0.001	0.003
	Concreteness	−0.0020	0.010	−0.18	0.857	0.893
	Semantic variability	−0.0074	0.010	−0.54	0.588	0.736

*Note*: Verbal fluency metrics are standardized on the mean and standard deviation of the whole sample.

Abbreviation: FDR, false discovery rate.

## 4 DISCUSSION

By leveraging item‐level analyses with fully automated computational techniques, this study seeks to refine diagnostic methodologies,[Table alz71124-tbl-0003] improve clinical applicability and scalability, and provide new insights into the cognitive and neural underpinnings of semantic and lexical processing in svPPA and lvPPA. We showed that an automated, item‐level analysis of verbal fluency responses robustly differentiates between matched groups of individuals with svPPA and lvPPA. Specifically, models incorporating word properties outperformed those relying solely on total correct responses or demographic and clinical variables, highlighting the translational potential of item‐level features often overlooked in standard scoring. Understanding these patterns could enhance theoretical models of semantic memory impairment and clinical assessment of PPA variants. Moreover, these findings support growing evidence advocating for more nuanced, multivariate approaches to assessing verbal fluency in neurodegenerative diseases.^15^, ^18^, ^28^

Relative to individuals with lvPPA, those with svPPA consistently produced words that were more general, shorter, more frequent, acquired earlier in life, and drawn from denser semantic and phonological neighborhoods—patterns suggesting reliance on more accessible words in the semantic memory network during retrieval.^50^ This tendency is predictable, given that svPPA is defined by profound semantic memory impairment, and aligns with findings in other conditions marked by semantic degradation.^15^, ^51^ These item‐level metrics better differentiated the two patient groups than the standard total correct score, which showed the smallest effect size in group comparisons. This finding may reflect that total correct is a composite outcome influenced by multiple cognitive systems—including attention, working memory, and processing speed—whereas word properties provide more direct insight into the semantic and phonological networks targeted in PPA. Importantly, these networks are differentially affected: lvPPA is characterized by lexical retrieval difficulties involving phonological form and semantic access—depending on whether atrophy is centered in the angular versus superior temporal/supramarginal gyri—while svPPA involves a core semantic memory degradation. Item‐level features may thus capture the nature of the language deficit rather than just output quantity, providing a more specific view of linguistic breakdowns distinguishing these syndromes.

Our results align with prior work showing that specific item‐level metrics (e.g., age of acquisition, lexical frequency) are more sensitive markers of semantic system degradation than word count.^22^, ^24^, ^26^ We extend this research by demonstrating that combining multiple word properties in a multivariate model offers substantial gains in classification accuracy—achieving an AUC of 0.86—even when adjusting for age, sex/gender, education, and disease severity. This performance is comparable to studies using other (automated) language tasks, such as spontaneous speech analysis,^52^ supporting the clinical utility of item‐level fluency metrics. Moreover, although the lvPPA group differed from controls on fewer metrics than svPPA, they still showed robust differences on several phonological and lexical features, including number of phonemes/syllables and phonological neighborhood density. These features may be particularly useful for detecting early disease effects and monitoring progression in lvPPA, as semantic deficits are less prominent. Notably, our results showed no role of semantic variability and concreteness in differentiating PPA groups or in associations with brain regions. This finding may be partially task related, as the semantic fluency task is constrained to animals, limiting variability in these word properties.

From a neurobiological perspective, most item‐level word properties were associated with gray matter volume in the left temporal pole and middle/superior temporal gyri, regions supporting semantic and lexical processing, respectively.^53^, ^54^, ^55^ While svPPA is marked by pronounced semantic impairment linked to characteristic temporal pole atrophy,^2^ both variants rely on overlapping temporal structures for word retrieval, particularly the middle/superior temporal gyri.^56^, ^57^ Although we hypothesized lexical features (phonological neighborhood density, syllable/phoneme length) would map onto lvPPA‐specific regions and semantic features onto svPPA‐specific regions, our results did not support this double dissociation. Instead, both lexical and semantic features converged in the left middle/superior temporal gyri—a region where both groups showed atrophy, and which did not differ significantly in gray matter volume between svPPA and lvPPA—and the temporal pole, which is svPPA specific. The lack of region‐specific correlations for word properties in lvPPA may reflect intercorrelations among word properties: words with few phonemes tend to also be more frequent and acquired earlier in life^58^ (as confirmed in our control‐group correlation matrix), potentially obscuring feature‐specific associations with posterior parietal regions. Notably, no item‐level properties were associated with lvPPA‐only regions (angular or supramarginal gyri); however, total correct responses was associated with the angular gyrus, as well as the temporal pole and middle temporal gyrus. This result may explain why total correct alone is less effective at differentiating subtypes: it reflects a combination of linguistic, executive, and working memory processes across a larger network,^59^ whereas item‐level word properties isolate more specific linguistic processes. It is also possible that feature‐specific correlations in lvPPA were harder to detect due to more variable and diffuse atrophy in posterior parietal regions compared to circumscribed anterior temporal degeneration in svPPA.^9^

Strengths of our study include the fully automated pipeline for extracting item‐level word properties, ensuring objectivity, reproducibility, and scalability—making this method suitable for clinical implementation and large‐scale studies. By examining a comprehensive set of word‐level features in one model, we provide a multidimensional view of verbal fluency performance that better reflects the complexity of underlying cognitive systems than univariate approaches. Another strength is our single‐center rare‐disease sample, including two large, well‐characterized and matched PPA subgroups—larger than most prior PPA literature^20^, ^60^, ^61^—allowing detection of group differences with smaller effect sizes. However, while our PPA groups are large for a rare disease cohort, the sample remains modest by statistical standards, limiting our ability to apply complex machine learning, k‐fold cross‐validation, or external validation. This limitation increases the risk of overfitting; however, consistent improvement in AUC values (a metric robust to marginal cases near the decision boundary) across five models when item‐level features are added, together with diagnostic label shuffling and an adequate feature‐to‐sample ratio, mitigate this concern. The confidence intervals around AUCs for the word‐property models reflect moderately wide ranges, but even lower bounds indicate good performance. Last, our sample was relatively highly educated and predominantly White, which may constrain generalizability. Future work should replicate findings in independent cohorts, ideally larger and more sociodemographically, linguistically, geographically, and culturally diverse populations.^62^

While svPPA is classically associated with semantic deficits and lvPPA with phonological impairment,^3^ accurate subtyping requires comprehensive assessment and expertise.^63^ In many clinical settings, however, lengthy language assessment batteries may not be feasible, and access to specialist evaluators (e.g., speech‐language pathologists) is limited. This situation underscores the value of objective and scalable tools that can enhance the information yield of existing tasks already embedded in standard care. Our findings show that a ubiquitous, 1 minute verbal fluency task—typically used to assess general language ability or executive function—can be computationally refined to reveal subtle linguistic and neuroanatomical patterns that distinguish PPA variants. By leveraging item‐level word properties, this approach offers a low‐burden, scalable framework to supplement standard assessments and better capture syndrome‐specific profiles in heterogeneous populations.^15^

A promising translational application is the development of an automated analysis tool that can serve as a screener to support diagnosis. Moreover, associations between word properties and left temporal lobe regions suggest that item‐level fluency metrics could serve as sensitive markers of disease progression, particularly in svPPA, in which anterior temporal lobe degeneration is a hallmark.^2^ Future longitudinal studies could investigate whether these features change earlier or more consistently than total correct scores, offering utility for monitoring disease course or therapeutic response. As a next step, incorporating automated transcription of audio‐recorded verbal fluency responses—instead of manually digitizing written responses—would further streamline the pipeline, enabling fully automated processing from raw audio to clinically actionable metrics. Additionally, our findings may inform theoretical models of semantic processing and neurodegenerative language disorders by elucidating the relationship between vocabulary structure and brain anatomy.

This study underscores the broader potential of automated, feature‐rich language analysis in clinical neuroscience. By moving beyond total correct scores and leveraging new computational capabilities of the digital era, we can uncover cognitive signatures more closely aligned with neural pathology within existing neuropsychological tests. As these analyses are fully automated and applicable to existing test data, they introduce no additional burden to patients or clinicians. Future research should extend this automated approach to other neurodegenerative syndromes with semantic deficits, including Alzheimer's disease, and assess its utility for early screening, staging, and intervention monitoring.

## CONFLICT OF INTEREST STATEMENT

Adolfo M. García is co‐founder of TELL. The authors report no further financial disclosures. Author disclosures are available in the supporting information.

## CONSENT STATEMENT

All participants or their caregivers provided informed consent in accordance with the Declaration of Helsinki. The study was approved by the UCSF Institutional Review Board (IRB #10‐03946) in accordance with the ethical standards as laid down in the 1964 Declaration of Helsinki and its later amendments.

## Supporting information



Supporting Information
